# Effects of Self-Esteem, Problem-Solving Ability, and Professional Nursing Values on the Career Identity of Nursing College Students in South Korea: A Cross-Sectional Study

**DOI:** 10.3390/ijerph18168531

**Published:** 2021-08-12

**Authors:** Jisun Min, Hyunlye Kim, Jaeyong Yoo

**Affiliations:** 1 Graduate Program in System Health Science and Engineering, Ewha Womans University, Seoul 03760, Korea; mjscodes91@naver.com; 2 Department of Nursing, College of Medicine, Chosun University, Gwangju 61452, Korea; jaeyongyoo@chosun.ac.kr

**Keywords:** career identity, self-esteem, problem-solving, professional nursing values, nursing students

## Abstract

In Korea, the number of admissions to nursing colleges has greatly increased over the past 20 years to address the shortage of nurses. However, many nursing students have unclear career identities during college and stop working in healthcare after graduation. This study aimed to examine the effects of self-esteem, problem-solving ability, and professional nursing values on career identity. The participants were 140 third- and fourth-year nursing students recruited from a university in South Korea. Data were collected between September and October 2019 using a self-administered questionnaire. Data were analyzed using descriptive statistics, *t*-test, ANOVA, Pearson’s correlation, and multiple linear regression. The results showed significant correlations between satisfaction with college life and major subject, subjective academic achievement, self-esteem, problem-solving ability, professional nursing values, and career identity. The factors that significantly affected career identity were self-esteem and professional nursing values. Nursing educators can support the career development of nursing students by enhancing their self-esteem and professionalism, along with efforts to improve satisfaction with their college life and major.

## 1. Introduction

The current shortage of nurses is a global health concern, especially during the coronavirus disease (COVID)-19 pandemic. Many countries are making efforts to improve nursing education and working conditions to overcome the undersupply of nurses. However, a more systematic solution is needed [1]. In South Korea over the past 20 years, the health policy of increasing the number of admissions to nursing colleges had led to an increased supply of licensed nurses. However, there is still a shortage of nurses in the field to such an extent that the number of working nurses per 1000 population remains below 50% of the OECD (Organization for Economic Cooperation and Development) average [2]. According to OECD statistics 2018, the level of nursing workforce production is at or above the OECD average, while the ratio of clinically working nurses to licensed nurses is about 34.5%, which is only half of the OECD average (65.3%) [3]. As this extremely low nurse retention rate is related to the high nurse turnover rate, active efforts from various angles are required [2,4]. In particular, the high resignation rate of new nurses suggests that nursing education can play a significant role in this problem.

The COVID-19 pandemic is not only a healthcare crisis, but also an opportunity to reshape the professional identity of nursing students [5]. A nationwide study conducted in China found that the professional identities of nursing students were strengthened during the COVID-19 pandemic [6]. Another study examined the professional identity and intention to leave the profession of nursing students during the COVID-19 pandemic. Participants with stronger professional identities intended to remain in nursing and reported an increase in enthusiasm during the pandemic [5]. Professional identity is important for the education, career development, and retention of students [7,8]. The career identity of college students was found to be closely related to occupational decision-making [9,10]. Therefore, career identity for nursing students is important to produce nurses who can contribute positively to healthcare.

Career identity is a component of self-image, and can be defined as a dynamic multiplicity of internal mental positions or “voices” regarding work [11]. Career identity is associated with a student’s competence, learning motivation and outcomes, and quality of choices [12]. The motivation, learning experiences, work performance, and career plans of nursing students are affected by their conceptualization of nursing practice and reasons for choosing the profession [13]. In a cross-sectional study of nursing students in South Korea, low nursing professionalism, such as a lack of understanding of the roles and responsibilities of nurses or having a negative occupational view, was associated with negative career-related identity and behavior [14]. Nursing educators should aim to enhance career identity to improve job satisfaction, and support nursing students in their career choices and planning. This study aimed to identify factors that influence career identity, and the results could be used to develop strategies to enhance career identity.

In previous studies of the career identity of nursing students, variables studied included self-esteem, problem-solving ability, professional values/professionalism, and learning experiences in nursing college. Several studies demonstrated that self-esteem correlated positively with the professional self-concept and values of nursing students [15,16]. In recent studies, self-esteem influenced the career identity of Korean nursing students [17,18,19]. Self-esteem also predicted the career preparation of nursing students [20]. Problem-solving ability was related to the maturity and motivation of Korean nursing students [21,22]. Problem-solving ability is one of the essential competencies for nursing students [23], but it is unclear how it relates to career identity. Another factor that may influence career identity is the conceptualization of the nursing profession. Professional values are the general behavioral principles that determine how well a profession suits an individual and their goals. These values are closely related to career choices [24]. In previous studies, professional values/professionalism were found to be related to career identity [14,25], and predicted the career plans of nursing students [26]. Finally, satisfaction with learning activities and academic achievement affected the career identity of nursing students in many previous studies [8,17,19,27,28]. 

**Figure 1:**
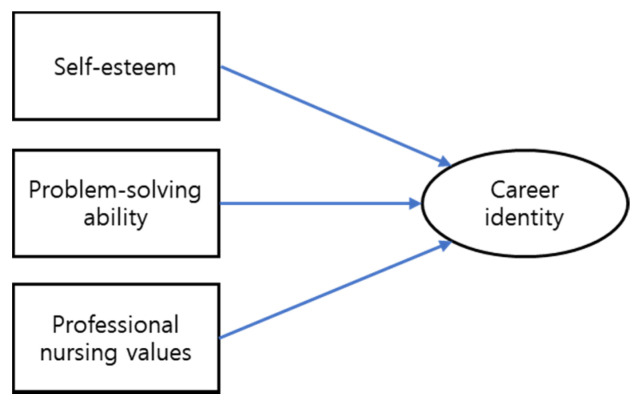
A hypothetical model for factors affecting career identity.

This study evaluated the career identity of nursing students, with the goal of formulating a nursing education strategy to produce nurses who can play an active role during healthcare crises subsequent to the COVID-19 pandemic. We aimed to evaluate the effects of self-esteem, problem-solving ability, and professional values on the career identity of nursing students. The hypothetical model of the variables analyzed in this study is shown in Figure 1.

## 2. Materials and Methods 

### 2.1. Samples

The sample of this study was nursing students with an average age of 21.84 years, who were mainly female. This cross-sectional descriptive study used convenience sampling. Students enrolled in a 4-year clinical nursing program were recruited from a university in G city. We included third- and fourth-year nursing students. The sample size required for regression analysis was calculated using G*Power software (version 3.1.9.2; Franz Faul, Universität Kiel, Germany). The required sample size was 119 for three predictors, with a medium effect size of 0.15, significance level of 0.05, and statistical power of 0.95. Only 6 of the 146 questionnaires were excluded because of incomplete responses or missing information, such that 140 were included in the final analysis.

### 2.2. Data Collection

Data were collected between September and October 2019. This study was approved by the institutional review board of C University (approval No.: 2-1041055-AB-N-01-2019-35; 4 September 2019). We explained the study objectives and procedures to the director of the nursing college and requested their cooperation. The survey was conducted directly by the first author in the classroom with the permission of the educator in charge. Participants were informed regarding the ethical principles of confidentiality, use of data for study purposes only, and freedom to withdraw from the study at any time. All participants gave written consent and completed the self-administered questionnaire, which took 15–20 min. The completed questionnaires were sealed in an envelope. The final enrolled participants were students in the third (56.6%) and fourth (43.4%) years of nursing colleges with similar proportions.

### 2.3. Variables

#### 2.3.1. General Characteristics

The questionnaire included questions about demographics (age, sex, and years of education), motives for enrolling in nursing college, satisfaction with college life, satisfaction with major subject at the time of admission to nursing college, current satisfaction with major subject, subjective academic achievement, desired career path after graduation, experiences related to college life, and major activities.

#### 2.3.2. Self-Esteem

Self-esteem was measured using the Korean version of the Rosenberg Self-esteem Scale [29,30]. This tool consists of 10 items rated on a five-point Likert scale (1–5: strongly disagree to strongly agree), with higher scores reflecting more positive feelings about the self. Items 3, 5, and 8–10 were reverse-scored. The internal consistency (Cronbach’s α) of this tool was 0.85 in a previous study [30] and 0.84 in the current study.

#### 2.3.3. Problem-Solving Ability

Problem-solving ability was assessed using the Problem Solving Scale developed by the Korea Educational Development Institute [31]. This tool is composed of nine sub-components with five items each: problem recognition, information collection, analysis, divergent thinking, decision-making, planning, execution and risk taking, performance evaluation, and feedback. This scale consists of 45 items rated on a five-point Likert scale (1–5: very rarely to very often), with higher scores reflecting greater problem-solving ability. The Cronbach’s α for this tool was 0.94 (0.63–0.77 for sub-components) [31] at the time of its development and 0.91 (0.58–0.81) in this study. An example of a scale item included in this instrument is as follows: “I first check what the problem is to be solved”.

#### 2.3.4. Professional Nursing Values

Professional nursing values were measured using the Nursing Professional Values Scale, which was developed in Korea and consists of 29 items [32] and five sub-domains: self-concept of the profession (nine items), social awareness (eight items), nursing professionalism (five items), roles of nursing service (four items), and nursing originality (three items). Each question is rated on a five-point Likert scale (1–5: not at all to very much), with higher scores reflecting greater nursing professionalism. Items 16, 20, and 24 were reverse-scored. The Cronbach’s α for this tool was 0.92 (0.53–0.86 for sub-domains) [32] at the time of its development and 0.89 (0.46–0.83) in this study. Examples of items on the scale included in this instrument are as follows: “Nurses are considered to be trusted by patients” and “Nurses are known to perform tasks independently and autonomously”.

#### 2.3.5. Career Identity

Career identity was measured using the identity subscale of the Korean version of the My Vocational Situation (MVS) scale [33,34], modified for Korean nursing students [34]. The scale consists of 14 items, rated on a four-point Likert scale (1–4: strongly disagree to strongly agree). The responses for all items, except question 6, were reverse-scored. Higher scores correlated with a greater sense of career identity. The Cronbach’s α for this tool was 0.88 in a previous study [35] and 0.87 in this study. An example of a scale item included in this instrument is as follows: “I’m not sure I’ll do well for my chosen nursing profession”.

### 2.4. Statistical Analyses

Data analyses were performed using SAS statistical software (version 9.4; SAS Institute, Cary, NC, USA). Descriptive statistics, including means, standard deviations, frequencies, and percentages, were used to describe the baseline characteristics and study variables. Differences in variables based on the general characteristics of participants were analyzed using independent *t*-tests and analysis of variance (ANOVA) with Scheffé post-hoc tests. The relationships between variables were analyzed using Pearson’s correlation coefficient. However, non-parametric tests were performed for self-esteem that was not normally distributed by the Shapiro‒Wilk test (*p* = 0.017) and the Q‒Q plot: Mann‒Whitney U test, Kruskal‒Wallis test with Bonfferoni post-hoc tests, and Spearman rank correlation. After the basic assumptions of regression analysis were assessed, multiple linear regression was used to determine the factors that predicted career identity.

## 3. Results

### 3.1. General Characteristics

The average age of the participants was 21.84 ± 1.24 years, and most participants were female (82.5%). The participants were enrolled in the third (56.6%) or fourth (43.4%) year of nursing college. The reasons cited for choosing nursing college were its high employment rate (46.2%), academic performance (20.4%), and interests and aptitudes (19.4%). Satisfaction with college life was reported by 41.4% of participants, and satisfaction with the major subject was reported by 41.4% of participants at the time of admission and 42.1% currently. In terms of subjective academic achievement, a significant proportion of participants (65.0%) reported that they were at an intermediate level. Most participants (75.5%) wanted to work as a hospital nurse after graduation (Table 1).

### 3.2. Descriptive Statistics of Study Variables

Table 2 presents the descriptive statistics of the variables of interest and its subcomponents. The mean score was 3.80 ± 0.67 out of 5 for self-esteem, 3.54 ± 0.38 out of 5 for problem-solving ability, 3.62 ± 0.44 out of 5 for professional nursing values, and 2.52 ± 0.55 out of 4 for career identity.

### 3.3. Differences in Variables According to General Participant Characteristics 

Table 3 presents the differences in the variables according to the participants’ general characteristics. There were statistically significant differences in self-esteem, problem-solving ability, professional nursing values, and career identity according to college life satisfaction, satisfaction with major subject, and subjective academic achievement. In addition, self-esteem differed significantly between grades, and career identity differed between sexes and grades.

### 3.4. Correlations between Variables

Spearman’s correlation analysis and Pearson’s correlation analysis showed that career identity had a significantly positive correlation with self-esteem (Spearman’s rho = 0.49, *p* < 0.001), problem-solving ability (r = 0.31, *p* < 0.001), and professional nursing values (r = 0.37, *p* < 0.001). In addition, professional nursing values positively correlated with self-esteem (Spearman’s rho = 0.30, *p* < 0.001) and problem-solving ability (r = 0.44, *p* < 0.001). Problem-solving ability was positively correlated with self-esteem (Spearman’s rho = 0.42, *p* < 0.001) (Table 4).

### 3.5. Factors Affecting Career Identity

In the initial regression model, self-esteem, problem-solving ability, and professional nursing values were set as independent variables. Considering the high significance in the correlation between independent variables, a regression diagnosis was made to identify the problem of endogeneity. All correlation coefficients were less than 0.5 (0.30–0.49), tolerances were more than 0.1 (0.76–0.89), and variance inflation factor (VIF) was less than 10 (1.12–1.32). However, low eigenvalues (0.009), a high condition index (21.114), and simultaneously a high proportion of variance (31.8%; 77.6%) were observed in self-esteem and professional nursing values, indicating collinearity. After removing the problem-solving ability to explain less the dependent variable among these, multiple regression analysis was then performed.

Table 5 shows the results of multiple regression analysis, with self-esteem and professional nursing values as independent variables. The Durbin‒Watson value was 1.749, indicating no auto-correlation. The tolerances were more than 0.1 (0.93), and the VIF was less than 10 (1.07), indicating no multicollinearity. The regression model was fit (F = 32.89, *p* < 0.001), and the explanatory power was 31.5%, slightly higher than that (31.0%) of the initial model. The factors affecting career identity were self-esteem (β = 0.450, *p* < 0.001) and professional nursing values (β = 0.252, *p* = 0.001).

## 4. Discussion

This study presented significant associations with college life and career-related characteristics, self-esteem, problem-solving ability, professional nursing values, and career identity of Korean nursing students. In particular, it was confirmed that self-esteem and professional nursing value were predictors of career identity. 

First, the characteristics of the participants’ college life and career are as follows. In this study, many nursing students reported that they were motivated to study nursing because of the high employment rates, and many wanted to work in a general hospital after graduation. These findings were similar to those of a study conducted by Seong et al. (2012) in Korea [14]. However, nurses in many countries, including Korea, often stop working as clinical nurses after graduation. Healthcare crises similar to the COVID-19 pandemic may occur again in the future. Nursing educators should help nursing students who wish to become professional nurses by playing an active role as guardians to promote a better healthcare environment.

The self-esteem of our participants (rated as 3.80 out of 5 points overall) was significantly higher among the fourth-year (4.02) compared with third-year students (3.62). Similar results were reported by a descriptive longitudinal study conducted in Turkey that explored the effects of a 4-year nursing college educational program on self-esteem [36]. Self-esteem was higher among students satisfied with their college life and major subject, and among those with higher subjective academic achievement, consistent with the results of earlier studies [19,28,37,38,39,40,41]. Educational interventions to strengthen self-esteem should help to ensure that nursing students are satisfied with their college life and major subjects, and achieve high academic performance.

In this study, problem-solving ability (rated as 3.54 out of 5 points) correlated positively with satisfaction with college life and the major subject, and with academic achievement, consistent with the results of previous studies of Korean nursing students [21,41,42,43]. Luo et al. (2019) emphasized the importance of the problem-solving skills of nursing students in the context of self-directed learning, because nurses must be able to respond quickly using reasoning [23]. Nursing education curricula should be based on problem-solving instead of rote-learning to enhance knowledge accumulation.

In this study, the professional nursing values (rated as 3.62 out of 5 points overall) with the lowest scores were social awareness (3.27) and nursing originality (3.58). This pattern was consistent with the results of a study by Kim & Kim (2019), who used the same tools for assessing Korean nursing students [44]. These results suggest that efforts are needed to enhance social awareness among nurses and emphasize the uniqueness of nursing, in order to enhance the professional intuition of nursing students. In addition, professional nursing values differed significantly by college life satisfaction, satisfaction with the major subject, and academic achievement. In many previous studies, scores for professional nursing values were higher among students who were satisfied with their university life or major subject, and among students with high academic achievement [44,45,46,47]. Poorchangizi et al. (2019) emphasized that the professional values of nursing students vary based on the educational strategy of their institution, and that these values should be enhanced by integrating them into the curriculum [48].

Career identity (rated as 2.52 out of 4 points) was significantly greater in our female students and students in the fourth year. Studies of differences in career identity among students according to sex and grade showed inconsistent results [18,49,50]. Career identity was relatively strong in our study population, as evidenced by high college life satisfaction, major subject satisfaction, and academic achievement. Similar results were found in many previous studies [10,27,49,50,51,52,53]. Nursing educators can help students establish their career identity through career development programs, and by adjusting the curriculum beginning in the junior year.

We observed positive correlations among self-esteem, problem-solving ability, professional nursing value, and career identity. Many previous studies have also reported correlations among these variables [14,17,18,19,25,28,39,42,43,54]. These factors could be used to establish an effective career development strategy and enhance career identity.

In multiple regression analysis, self-esteem and professional nursing values predicted career identity. An effect of self-esteem on career identity has also been identified in previous studies [17,19,28]. According to a qualitative study of young Australians, subjective achievement affected the formation of career identity [55]. Therefore, a career development program with activities and experiences promoting positive self-evaluation may be effective. Previous studies have reported effects of professional nursing values among nursing students on career identity [53], and of nursing professionalism on career plans [14]. In addition, a qualitative study of career identity among Vietnamese nursing students reported that personal values, needs, plans regarding future career, and sociocultural values significantly affected career identity [56]. A study of the professional identity of U.K. nursing students using focus group interviews demonstrated that the students had “dualistic” attitudes, expressing both idealism and cynicism; they also expressed anxiety about nursing work, and perceived nurses to be in a “powerless position” within the professional healthcare hierarchy [57]. These results suggest that individual nursing values and perceptions of the realities of nursing have important influences on career identity and future career paths. Nursing educators should help nursing students understand how professional nursing values contribute to a healthy life, and help them to formulate career plans.

A major limitation of this study is the difficulty of generalization owing to the small sample size from a single university. Future studies should include larger and more diverse samples, and assess different interventions to definitively determine the factors that most affect career development.

## 5. Conclusions

In this study, self-esteem and professional nursing value were factors influencing career identity, along with their association with college life/major satisfaction and problem-solving ability. Nursing educators need to devise a career development program that can enhance students’ self-esteem and professionalism, along with efforts to improve satisfaction with their college life and major. Health officials should support the establishment of a quality education and career development system for expanded nursing college students. These proactive measures would help nurses to lead a satisfactory college life, develop nursing competencies, and be motivated to continue to work in healthcare after graduation.

## Figures and Tables

**Table 1:** General characteristics of the participants (*N* = 140).

Variable	Category	*n* (%) or M ± SD
Age (y)		21.81 ± 1.22
Sex	Female Male	116 (82.9%) 24 (17.1%)
Grade	3rd year 4th year	78 (55.7%) 62 (44.3%)
Motives for choosing nursing college *	High employment rate Academic performance Recommendations from others Family wishes Personal interests and aptitudes Other	67 (46.5%) 30 (20.8%) 7 (4.9%) 8 (5.6%) 28 (19.4%) 4 (2.8%)
College life satisfaction	Satisfied Normal Unsatisfied	58 (41.4%) 65 (46.4%) 17 (12.2%)
Satisfaction with major subject on admission	Satisfied Normal Unsatisfied	59 (42.1%) 68 (48.6%) 13 (9.3%)
Current satisfaction with major subject	Satisfied Normal Unsatisfied	57 (40.7%) 70 (50.0%) 13 (9.3%)
Subjective academic achievement	High Middle Low	29 (20.7%) 91 (65.0%) 20 (14.3%)
Desired career path after graduation *	General hospital Public officer Health teacher Overseas employment Other	108 (75.5%) 26 (18.2%) 3 (2.1%) 4 (2.8%) 2 (1.4%)

M = mean; SD = standard deviation; *n* = number of participants. * Includes duplicate responses.

**Table 2:** Descriptive statistics for variables (*N* = 140).

Variables (Number of Items)	M ± SD	Minimum	Maximum	Possible Range
1. Self-esteem (10)	3.80 ± 0.67	1.50	5.00	1–5
2. Problem-solving ability (45) (2-1) Problem recognition (5) (2-2) Information collection (5) (2-3) Analysis (5) (2-4) Divergent thinking (5) (2-5) Decision-making (5) (2-6) Planning (5) (2-7) Execution and risk taking (5) (2-8) Evaluation (5) (2-9) Feedback (5)	3.54 ± 0.38 3.74 ± 0.48 3.42 ± 0.55 3.77 ± 0.62 3.07 ± 0.51 3.83 ± 0.53 3.50 ± 0.70 3.23 ± 0.59 3.59 ± 0.50 3.69 ± 0.55	2.13 2.60 2.40 1.80 1.80 2.60 1.00 1.20 2.40 2.00	4.64 5.00 5.00 5.00 4.40 5.00 5.00 5.00 5.00 5.00	1–5
3. Professional nursing values (29) (3-1) Self-concept of the profession (9) (3-2) Social awareness (8) (3-3) Nursing professionalism (5) (3-4) Roles of nursing service (4) (3-5) Nursing originality (3)	3.62 ± 0.44 3.73 ± 0.49 3.27 ± 0.63 3.84 ± 0.46 3.86 ± 0.52 3.58 ± 0.59	2.24 2.67 1.13 2.60 2.00 2.00	4.66 5.00 4.75 5.00 5.00 5.00	1–5
4. Career identity (14)	2.52 ± 0.55	1.36	3.79	1–4

M = mean; SD = standard deviation; *n* = number.

**Table 3:** Differences in variables according to the participants’ general characteristics (*N* = 140).

	Self-Esteem			Problem-Solving Ability		Professional Nursing Values		Career Identity	
	Median (IQR)	Mean Rank	U/χ^2^_kw_ (df) (*p*-Value) Bonfferoni Test	M ± SD	F/t (*p*-Value) Scheffé Test	M ± SD	F/t (*p*-Value) Scheffé Test	M ± SD	F/t (*p*-Value) Scheffé Test
Sex									
Female Male	3.90 (0.975) 3.75 (1.250)	72.39 61.35	1172.50 (0.113)	3.56 ± 0.38 3.42 ± 0.38	1.74 (0.085)	3.65 ± 0.45 3.49 ± 0.40	1.70 (0.092)	2.56 ± 0.56 2.32 ± 0.45	2.02 (0.045)
Grade									
3rd year 4th year	3.60 (0.925) 4.10 (0.850)	59.92 83.81	1592.50 (<0.001)	3.49 ± 0.38 3.60 ± 0.37	−1.64 (0.103)	3.56 ± 0.43 3.70 ± 0.45	−1.88 (0.062)	2.31 ± 0.47 2.78 ± 0.53	−5.49 (<0.001)
College life satisfaction									
Satisfied ^a^ Normal ^b^ Unsatisfied ^c^	4.20 (0.900) 3.60 (1.000) 3.70 (1.100)	90.80 58.04 48.88	24.514 (2) (<0.001) a > b, c	3.70 ± 0.37 3.47 ± 0.32 3.25 ± 0.41	12.87 (<0.001) a > b, c	3.78 ± 0.44 3.56 ± 0.43 3.35 ± 0.29	8.37 (<0.001) a > b, c	2.85 ± 0.54 2.34 ± 0.41 2.11 ± 0.43	25.59 (<0.001) a > b, c
Major satisfaction at admission									
Satisfied ^a^ Normal ^b^ Unsatisfied ^c^	4.20 (0.900) 3.70 (0.850) 3.00 (0.750)	86.91 63.58 35.81	24.711 (2) <0.001 a > b > c	3.67 ± 0.33 3.42 ± 0.39 3.52 ± 0.38	6.82 (0.002) a > b	3.75 ± 0.46 3.56 ± 0.40 3.41 ± 0.43	4.81 (0.010) a > b, c	2.88 ± 0.49 2.32 ± 0.38 1.90 ± 0.44	40.20 (<0.001) a > b > c
Satisfaction with current major									
Satisfied ^a^ Normal ^b^ Unsatisfied ^c^	4.40 (0.550) 3.70 (0.900) 3.40 (1.175)	101.07 64.81 52.08	16.399 (2) (<0.001) a > b, c	3.69 ± 0.33 3.46 ± 0.36 3.32 ± 0.48	9.19 (<0.001) a > b, c	3.77 ± 0.45 3.57 ± 0.41 3.28 ± 0.32	8.57 (<0.001) a > b, c	2.92 ± 0.44 2.31 ± 0.42 1.92 ± 0.38	46.88 (<0.001) a > b > c
Subjective academic achievement									
High ^a^ Middle ^b^ Low ^c^	4.20 (0.800) 3.65 (0.900) 3.40 (1.700)	88.28 57.74 56.58	19.732 (2) (<0.001) a > b, c	3.70 ± 0.31 3.54 ± 0.37 3.30 ± 0.41	7.02 (<0.001) a, b > c	3.87 ± 0.35 3.61 ± 0.44 3.36 ± 0.39	8.79 (<0.001) a > b, c	2.79 ± 0.46 2.51 ± 0.52 2.17 ± 0.59	8.34 (<0.001) a, b > c

M = mean; SD = standard deviation; IQR = interquartile range; In the variables of College life satisfaction, Major satisfaction at admission, Satisfaction with current major, “a” is the Satisfied category, “b” is the Normal category, and “c” is the Unsatisfied category. In the Subjective academic achievement variable, “a” is the High category, “b” is the Middle category, and “c” is the Low category.

**Table 4:** Correlations between variables (*N* = 140).

Variable	1	2	3	4
	Spearman’s Rho (*p*-Value)	r (*p*-Value)		
1. Self-esteem	1			
2. Problem-solving ability	0.42 (<0.001)	1		
3. Professional nursing values	0.30 (<0.001)	0.30 (<0.001)	1	
4. Career identity	0.49 (<0.001)	0.31 (<0.001)	0.37 (<0.001)	1

**Table 5:** Factors that affect career identity (*N* = 140).

Variables	B	SE	β	t	*p*
Self-esteem	0.369	0.059	0.450	6.19	<0.001
Professional nursing values	0.311	0.090	0.252	3.47	0.001
R^2^ = 0.324, adjusted R^2^ = 0.315, F = 32.89, *p* < 0.001					
